# Organ function biomarker abnormalities, associated factors and disease outcome among hospitalized patients with COVID-19

**DOI:** 10.2217/bmm-2021-0681

**Published:** 2022-03-02

**Authors:** Shambel Araya, Yakob G Tsegay, Assegdew Atlaw, Mintsnot Aragaw, Getachew Tadlo, Nebiyu Tsegaye, Daniel Kahase, Zenebe Gebreyohanes, Mollalegne Bitew, Nega Berhane

**Affiliations:** ^1^Addis Ababa University College of Health Science, Department of Medical Laboratory Science, Addis Ababa, Ethiopia; ^2^Department of Medical Laboratory Science, St. Paul Hospital Millennium Medical College (SPHMMC), Addis Ababa, Ethiopia; ^3^Department of Medical Biotechnology, Institute of Biotechnology, University of Gondar, Gondar, Ethiopia; ^4^Department of Research & Development Center, College of Health Sciences, Defense University, Addis Ababa, Ethiopia; ^5^Addis Ababa University, College of Health Science, Department of Medical Microbiology, Immunology & Parasitology, Addis Ababa, Ethiopia; ^6^Biotechnology Institute, Addis Ababa, Ethiopia; ^7^Department of Medical Laboratory Sciences, College of Medicine & Health Sciences, Wolkite University, South Nation Nationality & Peoples, Ethiopia

**Keywords:** Addis Ababa, cardiac marker, co-morbidity, COVID-19, Ethiopia, liver function tests, mortality, renal function tests

## Abstract

**Background:** The aim of this study was to determine the magnitude of abnormal organ function tests and biomarkers in hospitalized patients with confirmed COVID-19 and to define the association among markers of organ failure, disease severity and its outcome in hospitalized COVID-19 patients in Ethiopia. **Methods:** A prospective cohort study was conducted among COVID-19 patients admitted to Millennium COVID-19 Treatment Center from December 2020 to June 2021. **Results:** The median age of the 440 study participants was 60.3 ± 1.3 years, and from these 71.3% of patients were male. Disease severity: p-value: 0.032; adjusted odds ratio (AOR) (95% CI): 4.4 (0.022–0.085); and the presence of any co-morbidity; p-value: 0.012; AOR (95% CI): 0.80 (0.47–0.83) was significantly associated with mortality. Aspartate transaminase, alanine transaminase and alkaline phosphatase parameter values of patients overall, were elevated – mainly among critical patients (56.9 ± 57.7, 58.5 ± 63 and 114.6 ± 60, respectively).

SARS-CoV-2 primarily causes pneumonia, and the disease was named COVID-19 [1,2]. COVID-19 symptoms are highly variable, ranging from severe illness to none. It can spread mainly through the air when people are in close contact with or near each other. Human-to-human transmission is the main method of infection, and it can spread via contaminated surfaces [3,4]. The infection is characterized by mild symptoms for most patients but for some individuals requires admission to intensive care [5,6]. People remain infectious for up to 2 weeks and can spread the virus even if they do not show symptoms [7–9].

Reports have shown that non-survivors of COVID-19 were more often older and men, and they had hyperlipidemia, chronic heart disease, a higher prevalence of diabetic mellitus, hypertension, a history of tobacco use and chronic obstructive pulmonary disease [1,3,10,11].

COVID-19 is characterized by acute respiratory failure and diffuse alveolar damage to the lungs [12], and the involvement of other organs was also mentioned by different authors [13–16]. After SARS-CoV-2 infects the lungs, the virus may migrate to the blood, accumulate in the kidneys and cause damage to resident renal cells [17,18]. Reports showed that 6.7–11.4% of patients with SARS-CoV-2 results acute kidney injury and the mortality of those with acute kidney injury was 80–91.7% [19–21]. During COVID-19 infection, multiple organs, including the heart, kidney and liver, can be affected, depending on the state and severity of the infection [22,23].

COVID-19 can also cause heart muscle injury, as determined by the release of high-sensitivity troponin [3,24]. The occurrence of high cardiac-specific structural protein in the serum is associated with severe disease progression. In addition to this, blood clotting can be impaired as a result of systemic inflammation from the infection [24–26].

Vast amounts of information was obtained on COVID-19 clinical features, but limited information has been provided on organ function laboratory biomarker abnormalities, the association of disease severity and organ function test parameters and disease outcome from an Ethiopian perspective. Therefore, the aim of this study was to determine the magnitude of abnormal organ function test parameters in hospitalized patients with confirmed SARS-CoV-2 infection and to define the association between markers of organ failure and disease severity and its outcome in patients infected with SARS-CoV-2 in Ethiopia.

## Methodology

### Ethical consideration

The study was approved by the Institute of Biotechnology, University of Gondar, ethics and research committee, protocol number IOB/291/04/2021. Written informed consent was obtained from the study subjects. Data were collected after official permission was obtained from the treatment center. All the information obtained from the study participants was kept confidential.

## Study design & setting

An institution-based prospective cohort study was conducted at Millennium COVID-19 Care Center, a makeshift hospital in Addis Ababa, Ethiopia. The center is remodeled from the previous Millennium Hall, which was a recreational center. The center started accepting reverse transcription PCR (RT-PCR)-positive patients irrespective of the presence of symptoms on 2 June 2020, with a capacity of 1000 beds, 40 intensive care (ICU) beds and 12 mechanical ventilators. Angiotensin-converting enzyme inhibitors (ACEIs), angiotensin receptor blockers (ARBs) and NSAIDs were used to treat COVID-19 in the center. Of the total study participants, 13.5% had a history of ARB, ACEI and NSAID use. Among the patients in the age group >56 years, a significant proportion had a history of taking ACEIs, ARBs and NSAIDs. Drugs such as dexamethasone were also used in the center for COVID-19 treatment [27]. Therefore, the center was used as both a quarantine and a treatment center in order to halt transmission of the disease.

The center was designed to admit adult and non-pregnant patients only, but since the other centers were not ready for COVID-19 case admission, for several months all age groups and pregnant patients were also being admitted. The center accepts patients from referral centers (health center up to tertiary hospital which doesn't have a COVID-19 care setup) only if they present a positive RT-PCR result, and the service is provided for free.

### Sample collection & clinical chemistry tests analysis

This study collected 440 clinical histories, sociodemographic characteristics and laboratory investigation results from the laboratory database of Millennium COVID-19 Care and Treatment Center, Addis Ababa, Ethiopia, from December 2020 to June 2021. Requests to the laboratory are generated online, and the laboratory results are sent electronically from the laboratory information system to the patient's electronic medical record.

This study investigated the profiles of the clinical chemistry tests of COVID-19 RT-PCR-confirmed hospitalized cases using a routine clinical chemistry automated analyzer. Patients who had been tested for biomarkers of liver function tests, renal function tests and cardiac marker tests were included in this study. Eight milliliters of venous blood were collected in serum separation gel tubes (SSGTs) for clinical chemistry test parameters. The liver function markers, renal function tests and cardiac markers were analyzed using the COBAS 6000 automated clinical chemistry analyzer. In most COVID-19 infections, RT-PCR tests confirmed cases; routine clinical chemistry tests were performed to assess renal, cardiac and liver function. Among these organ function biomarkers and tests, alanine transaminase (ALT), alkaline phosphatase (ALP) and aspartate transaminase (AST) were included under liver function tests, whereas creatinine kinase-MB (CK-MB) iso-enzymes, troponin and AST were included under cardiac function tests. In addition, creatinine and urea were mentioned as parameters for renal function tests. All clinical laboratory tests and interpretations were done following the manufacturers' recommendations and standard operating procedure. Liver test abnormalities were defined as an elevation of the following liver enzymes in serum above the normal range: ALT 0–33 U/l, AST 10–35 U/l and alkaline phosphatase 45–87 U/l. Renal function test abnormalities were defined as the elevation of creatinine value than the normal range (0.5–0.9 mg/dl) and urea (10–45 mg/dl). Cardiac biomarker abnormalities were defined as the elevation of troponin-T value than the normal range (0–14 pg/ml) and CK-MB (0–25 U/l).

## COVID-19 detection

SARS-CoV-2 was confirmed using RT-PCR. Two pairs of primers targeting the nucleo-capsid protein (N) and open reading frame 1ab (ORF1ab) were amplified and examined. The corresponding sequences for N were 5′GGGGAACTTCTCCTGCTAGAAT3′ (F), 5′CAGACATTTTGCTCTCAAGCTG3′(R) and 5′FAM-TTGCTGCTGCTTGACAGATTTAMRA3′ (probe) and for ORF1ab were 5′CCCTGTGGGTTTTACACTTAA3′ (F), 5′ACGATTGTGCATCAGCTGA3′ (R), and 5′-CY3 CCGTCTGCGGTATGTGGAAAGGTTATGGBHQ1-3′ (probe). These diagnostic criteria were based on recommendations from the WHO.

### Statistical analysis

SPSS statistical software package version 25.0 (SPSS, Inc., IL, USA) was used for statistical analysis. An adjusted odds ratio, 95% CI test was used to determine an association among variables such as age, sex, co-morbidities with disease severity. The quantitative data were expressed as mean ± standard deviation (SD) and median values. p < 0.05 was considered to be statistically significant.

## Operational definitions

### Moderate cases

Moderate cases comprise of symptomatic patients meeting the case definition for COVID without evidence of viral pneumonia or hypoxia. This may also include adolescents or adults with clinical signs of pneumonia (fever, cough, dyspnea, fast breathing) but without severe pneumonia, including SpO_2_ ≥90% on room air [28].

### Severe cases

Severe cases comprise of adolescents or adults with clinical signs of pneumonia (fever, cough, dyspnea, fast breathing) plus one of the following: respiratory rate >30 breaths/min, severe respiratory distress or SpO_2_ <90% on room air [28].

### Critical cases

Critical cases are identifiable by an acute respiratory distress syndrome (ARDS) within 1 week of known clinical insult or new or worsening respiratory symptoms, with chest imaging indicating bilateral opacities that cannot be fully explained by volume overload, labor or lung collapse; respiratory failure that cannot be fully explained by cardiac failure or fluid overload, acute life-threatening organ dysfunction, and evidence of septic shock with characteristics of persistent hypotension despite volume resuscitation in adults and children [28].

### Elevated result

Is a result higher than the normal reference range.

## Results

### Patient demographics & clinical features

A total of 440 patients with RT-PCR-confirmed COVID-19 disease were included in this study from a laboratory database. The median age of the study participants was 60.3 + 1.3 years; of these, 61.3% were male. Most of the study participants were older than 55 years (290/440 [65.9%]). The median age was significantly higher in the critical group (ICU patients) compared with the moderate and severe groups. Sixty-two of the 440 study participants had critical disease, and 306 of 440 had severe disease (Table 1). The duration of symptoms after admission was also higher in severe patients.

**Table 1.** Sociodemographic characteristics and clinical features of study participants at MCTC, Ethiopia, 2020.

Variables		Frequency	%
Gender	Male	270	61.3
	Female	170	38.7
Age	<55	150	33.1
	>55	290	65.9
Disease severity	Moderate	72	16.4
	Severe	306	69.5
	Critical	62	14.1
Co-morbidity	Yes	142	32.2
	No	298	67.8
Outcome	Survivor	352	80
	Non-survivor	88	20

**Figure 1.**
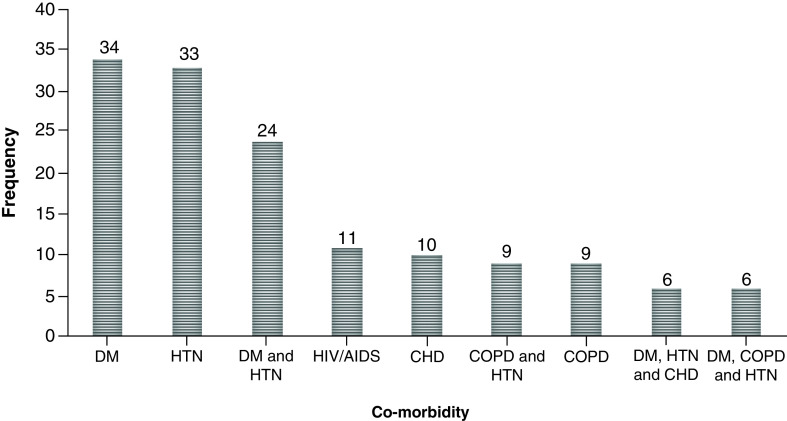
Distribution of co-morbidity among hospitalized COVID-19 patients.

More than one-third 142/440 (33.9%) of the patients had at least one co-morbidity. The major co-morbidities included diabetes mellitus (DM) (34/142 [24%]), hypertension (HTN) (33/142 [23.2%]) and HIV/AIDS (7/142) (4.9%). The total non-survivor rate in this study was 88/440 (20%). The mortality rate among individuals with at least one co-morbidity was 72/142 (50.7%; Figure 1).

In this study, 142/440 patients had at least one co-morbidity. Severe and ICU patients were more predisposed to death than moderate patients, and this was significantly associated with a p-value of 0.032. The presence of any co-morbidity was significantly associated with mortality (p = 0.012; AOR [95% CI]: 0.80 [0.47–0.83]). Even though it doesn't show a significant association, there was a high mortality rate among individuals older than 55 years (21% [61/290]; Table 2).

**Table 2.** Association of clinical history, sociodemographic characteristics and mortality among hospitalized COVID-19 patients, Addis Ababa, Ethiopia, 2021.

Variables			Survivor n = 352	Non-survivor n = 88	AOR	p-value
Age	<55 years		123	27	0.874 (0.491–1.556)	0.45
	>55 years		229	61	R	
Sex	Female		142	28	R	
	Male		210	60	0.56 (0.881–2.762)	0.86
Co-morbidity	No		297	1	R	
	Yes	DM	7	27	0.80 (0.47–0.83)	0.012
		HIV/AIDS	7	4		
		HTN	18	15		
		COPD	3	6		
		CHD	3	7		
		DM + HTN	14	10		
		COPD + HTN	3	6		
		DM + COPD + HTN	0	6		
		DM + COPD + CHD	0	6		
Disease severity	Moderate		66	7	R	
	Severe		271	34	3.7 (0.014–0.098)	0.041
	Critical		15	47	4.4 (0.022–0.085)	0.032

AOR: Adjusted odds ratio; CHD: Chronic heart disease; COPD: Chronic obstructive pulmonary disease; DM: Diabetes mellitus; HTN: Hypertension.

Liver function marker values of overall study participants were elevated, and three of these parameters, AST, ALT and ALP, were elevated among critical (ICU) patients (66.5 ± 65, 59.9 ± 58.7 and 120.6 ± 60, respectively). Creatinine, a renal function test, was more elevated among severe patients than those with moderate cases, whereas urea was more elevated among critical patients than moderate and severe patients. Troponin values of overall patients were elevated but CK-MB was in the normal range and the troponin value was markedly elevated among critical (ICU) patients. There was no marked difference among moderate, severe and critical groups for CK-MB values (p = 0.03; Table 3).

**Table 3.** Clinical chemistry test parameters among COVID-19-confirmed patients, Ethiopia, 2020.

Parameters	Normal range	Overall μ ± SD	Moderate μ ± SD	Severe μ ± SD	Critical μ ± SD	p-value
ALT (U/l)	0–33 U/l	59 ± 63	66.2 ± 87.4	45.28 ± 22.7	59.9 ± 58.7	>0.05
AST (U/l)	10–35 U/l	57 ± 53	64.35 ± 53	43.4 ± 28.4	66.5 ± 65	0.028
ALP (U/l)	45–87 U/l	112 ± 56	94.5 ± 36.53	103.4 ± 71.8	120.6 ± 60	0.092
Creatinine (mg/dl)	0.5–0.9 mg/dl	1.39 ± 6.5	0.93 ± 0.75	3.13 ± 14.36	0.96 ± 0.72	0.043
Urea (mg/dl)	10–45 mg/dl	44.8 ± 28.7	45 ± 29.45	37.7 ± 23	52 ± 31	0.058
Troponin-T (pg/ml)	0–14 pg/ml	60.1 ± 123.5	32.3 ± 89.65	35.7 ± 50	83 ± 143	0.0356
CK-MB (U/l)	0–25 U/l	3.3 ± 6.7	4.4 ± 11.07	1.39 ± 0.60	2.85 ± 2.91	0.003

ALP: Alkaline phosphatase; ALT: Alanine transaminase; AST: Aspartate transaminase; CK-MB: Creatinine kinase-MB; SD: Standard deviation.

Elevated AST (49.3%), ALT (62.3%), creatinine (37%) and troponin (59%) levels were seen among study subjects older than 55 years; 66.8% of males and 65% of ICU patients had elevated serum values of ALT and AST, respectively. Troponin was found elevated among males (54%) and among ICU (critical) patients (59%; Table 4).

**Table 4.** Frequency of laboratory profiles in relation to disease severity, age and sex among COVID-19 patients admitted to MCTC, Addis Ababa, Ethiopia, 2020.

Parameters		Age		Sex		Disease severity		
		<55 (%) n = 150	>55 (%) n = 290	Female (%) n = 170	Male (%) n = 270	Moderate (%) n = 72	Severe (%) n = 306	Critical (%) n = 62
ALT (U/l)	Elevated	56	62.3	44.4	66.8	53	59	65.4
AST (U/l)	Elevated	48.48	49.3	38	53.5	47.8	20.2	57
ALP (U/l)	Elevated	30	27.2	27	28.7	20.2	21.7	30.8
Creatinine (mg/dl)	Elevated	19.6	37	17.4	37.57	31.8	13	36.4
Urea (mg/dl)	Elevated	16.6	40.2	23.8	36.9	33.3	13	38.3
Troponin-T (pg/ml)	Elevated	37.8	59	47.6	54.7	39.13	36.2	59.8
CK-MB (U/l)	Elevated	30.3	28	34.9	26.1	14.4	27.5	31.7

ALP: Alkaline phosphatase; ALT: Alanine transaminase; AST: Aspartate transaminase; CK-MB: Creatinine kinase-MB.

## Discussion

COVID-19 is characterized by dysregulated immune responses, metabolic dysfunction and adverse effects on the function of multiple organs [22,23,26]. Even though this study did not find any significant association in outcomes by gender, males (61.1%) have a reasonably higher risk of being affected by COVID-19 than females (36.7%), and other reports [1,4,29] revealed similar findings.

Of all patients, 32.2% had at least one co-morbidity, including DM (34/142 [24%]), HTN (33/142 [23.2%]) and HIV/AIDS (7/142 [4.9%]). Different studies [1,4,30,31] also identified HTN, DM, COPD and viral infections, including HIV, hepatitis B and hepatitis C, as major co-morbidities associated with COVID-19 patients. These co-morbidities in the present study were also significantly associated with mortality rate (p = 0.032) and it could be explained by the fact that co-morbidity increases the severity of COVID-19 and prolongs the morbid condition. Another study, conducted in Sweden [32], also identified elderly patients as being more predisposed to COVID-19 disease severity, which is similar to the finding of the current study. This could be explained by the failure of the immune systems of older individuals with co-moribidities to defend against COVID-19 [33–35].

The laboratory parameter values indicate that, for COVID-19-positive cases, from routine clinical chemistry tests, such as liver function tests (ALT, AST and ALP), renal function tests (creatinine and urea) and cardiac function tests (troponin and ALP) were higher than the normal range.

Abnormal liver function marker results were seen in more than half of patients; 59% and 65.4% elevated ALT were found among critical and severe patients and 34.1% and 57% elevated AST among severe and critical patients were found, respectively. Different studies showed that COVID-19 stratification based on disease severity, including the need for ICU admission and the extent of respiratory distress, indicated that serum AST and ALT were elevated in patients with critical COVID-19 compared with those with severe and moderate disease [1,2,10,36–38]; during the 2002–2004 SARS outbreaks, this was also reported [39]. In COVID-19, the prognostic value of abnormal liver function tests is not well defined, but some studies state that abnormal liver function tests, particularly elevated AST, total bilirubin and ALT, are associated with increased disease severity [1,2,10,20,22,29,36–38], whereas other studies report that there is no association between disease severity and progression [40,41]. Even though the mechanism of the extrapulmonary spread of SARS-CoV-2, whether hematogenous, virus-mediated direct tissue damage or otherwise, remains elusive, different reports suggest that SARS-CoV-2 migration in the blood and intestines may infect the liver and damage hepatocytes, which could result in AST, bilirubin and ALT value elevation [42–44].

Secondary liver damage due to the administration of hepatotoxic drugs, respiratory distress syndrome-induced hypoxia, systemic inflammatory response and multi-organ failure are believed to be associated risk factors for COVID-19-related liver dysfunction [31,45].

Among renal function tests, serum urea and creatinine showed elevated values among severe (56.8%, 20.45%) and critical (59.8%, 36.4%) patients. Older age groups had elevated serum urea (40.2%) and creatinine (37%). Similar studies also revealed that renal function test alterations in COVID-19 patients was evidenced by increased serum urea and Creatinine values. Kidney tubular cells, which express the ACE2 receptor on their cellular surface, could be directly infected by SARS-CoV-2 [19,21]. Evidence also exists that kidney-resident cells can interact with circulating mediators, resulting in microcirculatory derangement, endothelial dysfunction and tubular injury [15,46,47].

Reports indicate that about 25–30% of individuals infected with SARS-CoV-2 develop acute kidney injury (AKI), which has been associated with increased mortality risk [15,48,49]. AKI occurred at much higher rates in critically ill patients admitted to hospitals, ranging from 78% to 90%, and it is a frequent complication of COVID-19 associated with mortality [50–52].

Cardiac injury is a common clinical feature of COVID-19 patients; this could result from SARS-CoV-2 infection as a result of direct and indirect effects on cardiomyocytes, including acute myocardial infarction, impaired renal function, heart failure, arrhythmias, myocarditis, sepsis, septic shock, cardiac arrest and pulmonary embolism [23,25,26]. In cardiac markers, CK-MB and troponin serum values were elevated among critical (59.8%), severe (56%) and moderate (39%) patients. A greater frequency and magnitude of troponin elevations in hospitalized patients have been associated with more-severe disease.

Petrosillo *et al.*, who compared the AST enzymes of different coronaviruses, found that an average elevated amount of up to 31.5% in SARS-CoV-2 cases compared with the other family of corona viruses [53]. This elevation of AST enzymes could be associated with cardiac problems associated with COVID-19, where AST together with CK-MB, lactate dehydrogenase (LDH) and troponin could be high. Underlying cardiac disease was reported as among the major risk factors in those susceptible to COVID-19 [24,54,55]. Myocardial injury, with elevation of cardiac biomarkers above the 99th percentile of the upper reference limit, occurred in 20–30% of hospitalized patients with COVID-19, with higher rates (55%) among those with pre-existing cardiovascular disease [56–58].

Guo *et al.* studied 187 COVID-19 patients, of whom 52 (27.8%) had a myocardial injury as determined by elevated levels of troponin [30]. The incidence of cardiac injury ranged anywhere between 8% and 12% in various studies, the incidence being 13-fold higher in the ICU/severe category [38,59,60]. Moreover, the patients admitted to the ICU had a 2.2-fold higher troponin level when compared with the non-ICU patients [59,60]. Therefore, cardiac damage biomarker evaluation upon admission and longitudinal monitoring during the hospital stay help to prompt intervention in order to improve the progression of the disease and could represent a significant tool for the early detection of cardiac injury [11,55]. In this study, the authors used a random sampling technique and there was a selection bias in the distribution of analytes across the severity grade where values were often closer to the moderate group. Outcomes in hospitalized patients could also have an inherent selection bias that hides the effect of sex.

## Conclusion

Co-morbidity and disease severity were significantly associated with mortality. Abnormal serum values of organ function biomarkers were associated with COVID-19 disease severity and a worse prognosis. Organ function biomarkers aid in risk stratification and the prediction of COVID-19 disease severity in order to guide clinical care. Among the biomarkers tested; AST, ALT, creatinine, urea, troponin and CK-MB were elevated parameters of organ function biomarkers of critical COVID-19. Organ function markers and tests should be monitored regularly for the management of COVID-19.

**Summary points**

- Co-morbidity and disease severity were significantly associated with mortality.
- Serum urea and creatinine showed elevated values among severe and critical COVID-19 patients.
- Older patients were more susceptible to COVID-19 disease severity.
- Troponin and creatinine kinase-MB range values were elevated among critical (intensive care [ICU]) patients in comparison to severe and moderate patients.
- Creatinine and urea values were higher among critical (ICU) patients than among severe and moderate patients.
- Aspartate transaminase, alanine transaminase and alkaline phosphatase values were higher among critical (ICU) patients than among severe and moderate patients.
- Organ function biomarkers aid in risk stratification and prediction of COVID-19 disease severity.
- Organ function markers and tests should be monitored regularly for the management of COVID-19.
